# An Unusual Lawn Mower Injury: A Case Report

**DOI:** 10.7759/cureus.77928

**Published:** 2025-01-24

**Authors:** Maxwell Davison-Kerwood, David M Langley, Alfredo Cordova, Ashley Grant

**Affiliations:** 1 Emergency Medicine, Florida State University College of Medicine, Sarasota, USA; 2 Trauma and Acute Care Surgery, Sarasota Memorial Hospital, Sarasota, USA

**Keywords:** battlefield medicine, emergency medicine and trauma, emergency medicine physician, general trauma surgery, high-velocity trauma, lawn mowing injuries, military trauma, penetrating abdominal trauma, point-of-care ultrasound, projectile removal

## Abstract

Penetrating abdominal injuries can be life-threatening and require prompt recognition and surgical intervention. We present the case of a 48-year-old male patient who presented to the emergency department with abdominal pain that began while mowing his lawn just before arrival but without any external signs of trauma. Point-of-care ultrasound revealed a possible linear hyperechoic foreign body just beneath the abdominal wall, a finding confirmed by computed tomography. The foreign body was subsequently retrieved in the operating room, and the patient had an excellent recovery. This type of abdominal injury mechanism is rarely documented in the literature. Relevant topics discussed include the incidence and burden of lawn mower injuries, their similarity to other projectile injuries, and potential challenges in diagnosing and managing such cases.

## Introduction

According to the research conducted by Harris et al., using the US Consumer Product Safety Commission's National Electronic Injury Surveillance System (NEISS) from 2005 to 2015, approximately 84,944 lawn mower injuries occur annually [1]. The most common causes of these injuries are direct contact with the blades or burns from hot mower parts.

Additionally, high-velocity objects can be ejected from the mower, presenting a complex clinical picture that requires thorough investigation. Objects such as wood, metal, or glass can cause severe injury and account for 3%-17% of all lawn mower injuries [1-3]. These injuries resemble battlefield "shrapnel-type" injuries [4]. Even today, since Russia's full-scale invasion of Ukraine, nearly one million casualties have occurred, with a significant proportion being shrapnel-type injuries from artillery, many resulting in amputations of the extremities [5]. This case contributes to the current body of literature by presenting a patient who experienced abrupt, severe pain while mowing his lawn but without known penetrating trauma. Initial examination revealed no external injuries, but further investigation revealed potentially catastrophic abdominal pathology requiring surgical intervention. This unique case highlights the similarity between lawn mower injuries and battlefield wounds, emphasizing the importance of a thorough physical examination, serial assessments, and awareness of potential diagnostic pitfalls for emergency physicians.

## Case presentation

**Figure 1:**
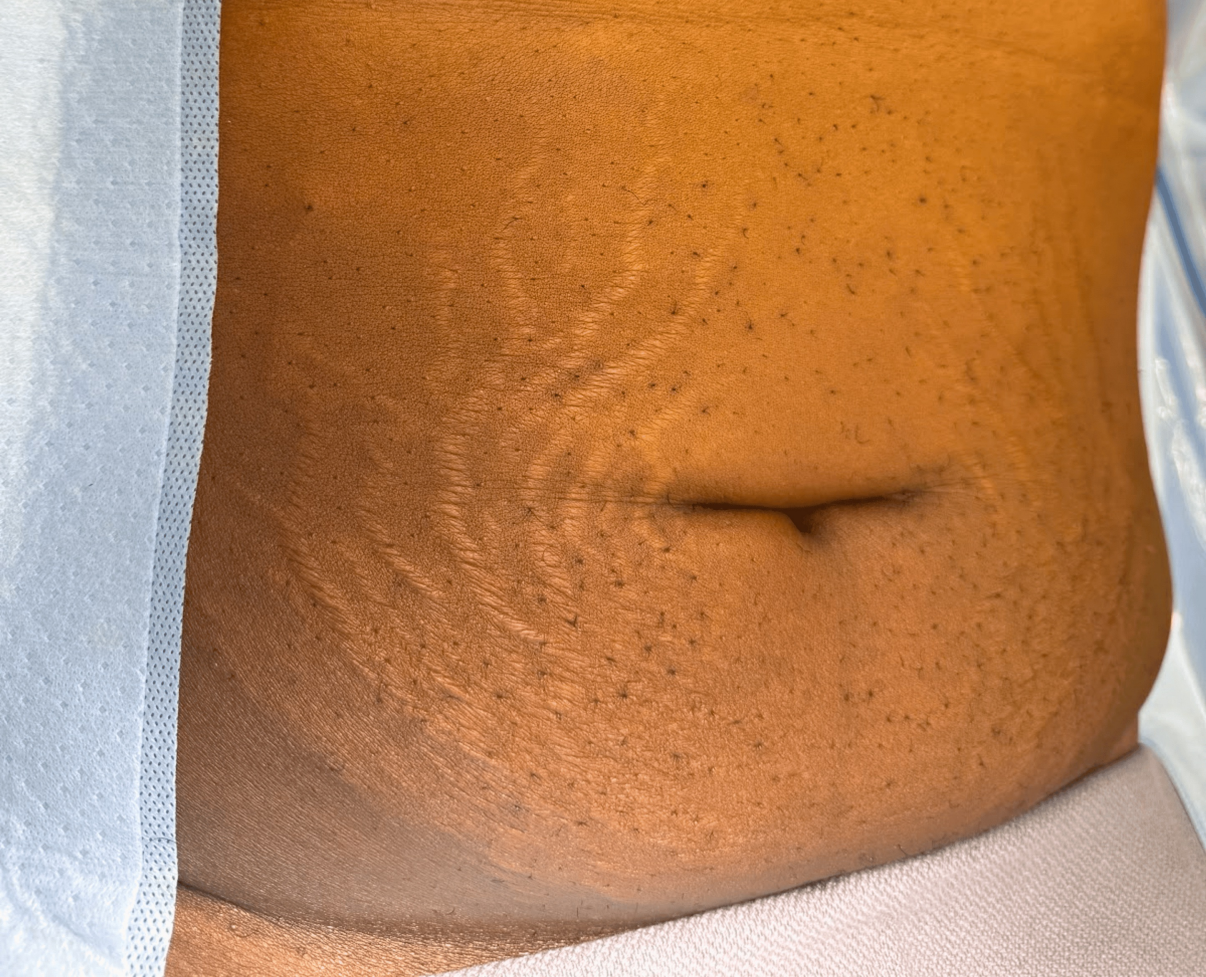
Image of the patient's abdomen.

A 48-year-old African American male patient with no significant past medical history presented to the emergency department with abdominal pain that began three hours prior while mowing his lawn. He initially believed he had been struck or stung while doing his chores but did not observe any apparent injury upon self-examination. Due to the persistence and severity of the abdominal pain, he sought further evaluation in the emergency department. On initial assessment, his vital signs were as follows: blood pressure 146/88 mmHg, respiratory rate 20 breaths per minute, heart rate 88 beats per minute, temperature 98.5 °F, and oxygen saturation of 100% on room air. Clinically, the patient appeared distressed due to pain, with tenderness to palpation in the right upper quadrant. His abdominal exam was otherwise unremarkable, showing no bruising or lacerations (Figure 1).

**Figure 2:**
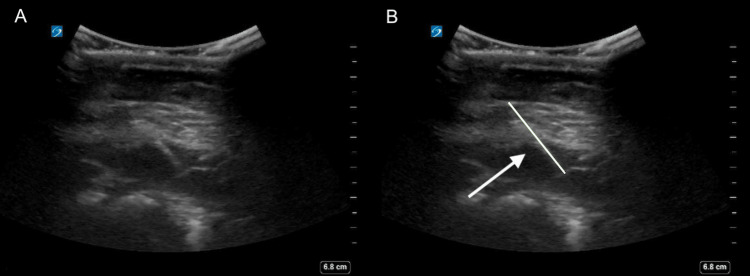
(A) POCUS image of the abdomen. (B) POCUS image of the abdomen with the linear, hyperechoic structure highlighted.

Due to the patient's presentation and concern for acute abdominal pathology, such as trauma with internal organ injury, acute cholecystitis, and choledocholithiasis, among others, a comprehensive workup including laboratory studies and computed tomography (CT) with intravenous (IV) contrast was ordered. In addition, a point-of-care ultrasound (POCUS) was performed to further investigate the possibility of free fluid in the abdomen and to evaluate for gallbladder pathology. The ultrasound was challenging due to the patient's discomfort and the presence of bowel gas. However, Figures 2A-2B reveal a possible hyperechoic linear structure just beneath the skin's surface.

**Figure 3:**
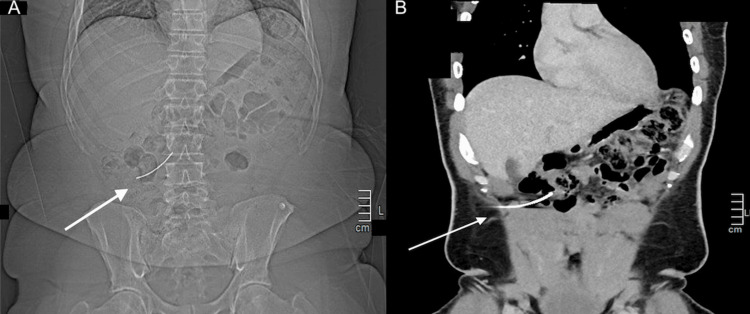
(A) Scout film before CT with the arrow pointing to the foreign body. (B) CT image with the arrow pointing to the foreign body.

Laboratory studies showed no acute abnormalities. The patient's white blood cell count was normal, with no significant anemia, and both lipase and liver enzyme levels were within normal limits. A CT scan confirmed the presence of a 7-cm metallic foreign body embedded within the abdominal cavity, without any radiographic evidence of bowel perforation, as seen on the scout image (Figures 3A-3B). 

Upon reexamination, the patient continued to report abdominal pain and discomfort. A slight inflammatory reaction was noted around the suspected site of injury in the right upper quadrant.

**Figure 4:**
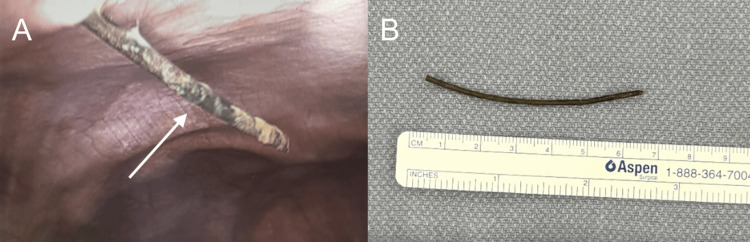
(A) Laparoscopic image of the wire. (B) Rusted silver wire measuring 7 cm post-retrieval.

It was unclear on CT whether the foreign body had punctured the bowel, but it was clear that it had violated the peritoneum. Trauma surgery was consulted, and after evaluation, they agreed with our assessment and proceeded with an emergent exploratory laparoscopy. Intraoperatively, they discovered a piece of rusty metal inside the peritoneal cavity (Figures 4A-4B). It appeared to be a nail and was removed without difficulty. To assess for bowel wall perforation, the bowel was examined from the cecum to the ligament of Treitz, and the colon was examined from the cecum to the sigmoid colon. No evidence of injury was found. The patient tolerated the procedure well and had an uneventful postoperative course and recovery. He was administered IV antibiotics intraoperatively, and his tetanus vaccination was updated. 

## Discussion

The novelty of this case report lies in the penetrating abdominal trauma caused by lawn mowing. Most lawn mower-related injuries are typically seen in the upper or lower extremities or face and generally involve lacerations, soft tissue injuries, or burns [6]. Clinicians should be aware that lawn mowers can produce high-velocity projectiles, leading to penetrating trauma from objects that would otherwise not be considered dangerous. Identifying such foreign bodies using ultrasound proved difficult, likely due to the object's angle relative to the beam angle (Figures 2A-2B). Fortunately, this foreign body was clearly identified on a CT scan (Figures 3A-3B). Physicians must be vigilant in the acute setting and consider imaging studies for patients with acute abdominal pain and suspected trauma, even when the physical examination appears reassuring, to avoid missing these injuries and ensure timely surgical intervention.

This patient presented with abdominal pain and initially believed an object had struck him, though he noted no apparent injury upon self-examination. This history prompted the physician to suspect a traumatic cause for his pain. However, it is conceivable that a physician may not obtain the entire history at initial presentation, as is the case with many intoxicated patients who fall and suffer occult traumatic injuries, or when a patient's doubts delay the recognition of trauma, creating a diagnostic challenge. An acute medical illness can sometimes present as a "precipitating cause" rather than a presumed traumatic injury. For example, lawn mowing-related leg pain was the presenting symptom in a 65-year-old patient who, after spending most of the day on a riding mower, presented with severe leg pain and was later diagnosed with aortic dissection [7]. Regardless of the clinical presentation, maintaining a low threshold for advanced imaging, such as CT scanning, is critical in identifying potentially serious injuries, particularly in cases of unexplained pain.

Overall, lawn mower injuries can be devastating, leading to a loss of function or life in the worst case, and are largely avoidable. One study found that a majority of injuries resulting from being struck by riding lawn mowers led to amputations [8]. From 2006 to 2013, the average emergency department cost for lawn mower injuries was roughly $14.1 million annually, with total inpatient charges for an average of $22.1 million annually [9]. Today's estimates would be approximately $20.3 million and $31.9 million adjusted for inflation annually, respectively [10]. These authors will undoubtedly consider this case for future complaints of abdominal pain after potential penetrating "shrapnel-like" trauma. This case report adds to the body of literature on this subject due to the critical injury caused by this small piece of debris, which is unusual as most injuries from lawnmowers are not penetrating abdominal trauma, as previously discussed. 

## Conclusions

The key learning points for evaluating a patient with a projectile lawn mower injury include performing a thorough physical exam and not being misled by a reassuring visual inspection. Although these injuries are uncommon, clinicians should maintain a low threshold for obtaining CT imaging to rule out potentially disastrous penetrating shrapnel-like injuries. 

To prevent lawn mowing injuries, operators should wear protective clothing, including steel-toed shoes, to safeguard against extremity injuries. Additionally, clearing the mowing area of rocks and debris can significantly reduce the risk of dangerous projectiles. The authors suggest that a warning sign near the ignition, reminding users to clear the area before mowing, could be an effective public health strategy to prevent such injuries.

## References

[1] Harris C, Madonick J, Hartka TR (2018). Lawn mower injuries presenting to the emergency department: 2005 to 2015. Am J Emerg Med.

[2] Lau ST, Lee YH, Hess DJ, Brisseau GF, Keleher GE, Caty MG (2006). Lawnmower injuries in children: a 10-year experience. Pediatr Surg Int.

[3] Mullins J (2010). Lawn mower injuries: a review. J Emerg Nurs.

[4] Rich NM (1967). Shrapnel wounds. JAMA.

[5] Sers R (2024). Ukrainian battlefield medicine. Lancet.

[6] Bachier M, Feliz A (2016). Epidemiology of lawnmower-related injuries in children: a 10-year review. Am J Surg.

[7] George N, Ganti L, Falgiani M, Desai B (2020). Aortic dissection presenting as leg pain. Cureus.

[8] Khansa I, Pearson GD, Bjorklund K, Fogolin A, Kirschner RE (2021). Pediatric lawnmower injuries: a 25-year review. JPRAS Open.

[9] Hottinger DG, Nasr I, Canner JK, Kattail D, Koka R, Schwengel D (2018). Incidence, distribution, and cost of lawn-mower injuries in the United States, 2006-2013. Public Health Rep.

[10] Inflation calculator. https://www.minneapolisfed.org/about-us/monetary-policy/inflation-calculator.

